# Association of Media Coverage of Transgender and Gender Diverse Issues With Rates of Referral of Transgender Children and Adolescents to Specialist Gender Clinics in the UK and Australia

**DOI:** 10.1001/jamanetworkopen.2020.11161

**Published:** 2020-07-28

**Authors:** Ken C. Pang, Nastasja M. de Graaf, Denise Chew, Monsurul Hoq, David R. Keith, Polly Carmichael, Thomas D. Steensma

**Affiliations:** 1 Department of Adolescent Medicine, Royal Children’s Hospital, Parkville, Australia; 2 Clinical Sciences Theme, Murdoch Children’s Research Institute, Parkville, Australia; 3 Department of Paediatrics, The University of Melbourne, Parkville, Australia; 4 Inflammation Division, The Walter and Eliza Hall Institute of Medical Research, Parkville, Australia; 5 Gender Identity Development Service, Tavistock and Portman NHS Foundation Trust, London, United Kingdom; 6 Department of Medical Psychology, Amsterdam UMC, Amsterdam, the Netherlands; 7 Center of Expertise on Gender Dysphoria, Amsterdam UMC, Amsterdam, the Netherlands; 8 Sloan School of Management, Massachusetts Institute of Technology, Cambridge

## Abstract

**Importance:**

Specialist gender clinics worldwide have witnessed an increase in referrals of transgender and gender diverse (TGD) children and adolescents, but the underlying factors associated with this increase are unknown.

**Objective:**

To determine whether increases in TGD young people presenting to specialist gender clinics are associated with related media coverage.

**Design, Setting, and Participants:**

This cross-sectional study was conducted at 2 publicly funded, pediatric specialist gender services, one located in the UK and the other in Australia. Participants were all children and adolescents aged 0 to 18 years, referred between January 1, 2009, and December 31, 2016, to their respective gender services in the UK and Australia. Data analysis was performed in April 2019.

**Exposures:**

Media coverage of TGD issues.

**Main Outcomes and Measures:**

Referral rates from each gender service were compared with local TGD-related media coverage during the study period.

**Results:**

Referral data for 5242 TGD young people were obtained (4684 in the UK, of whom 1847 [39.4%] were assigned male at birth and 2837 [60.6%] were assigned female at birth; 558 in Australia, of whom 250 [44.8%] were assigned male at birth and 308 [55.2%] were assigned female at birth), and a total of 2614 news items were identified (UK, 2194; Australia, 420). The annual number of TGD young people referred to both specialist gender clinics was positively correlated with the number of TGD-related local media stories appearing each year (Spearman *r* = 1.0; *P* < .001). Moreover, weekly referral rates in both the UK for week 1 (β̂ = 0.16; 95% CI, 0.03-0.29; *P* = .01) and Australia for week 2 (β̂ = 0.12; 95% CI, 0.04-0.20; *P* = .003) showed evidence of association with the number of TGD-related media items appearing within the local media. There was no evidence of association between referrals and media items appearing 3 weeks beforehand. Media predominantly focused on TGD issues showed some association with increased referral rates. Specifically, TGD-focused stories showed evidence of association with referral numbers at week 1 (β̂ = 0.16; 95% CI, 0.04-0.28; *P* = .007) and week 2 (β̂ = 0.23; 95% CI, 0.11-0.35; *P* < .001) in Australia and with referral numbers at week 1 (β̂ = 0.22; 95% CI, 0.01-0.44; *P* = .04) in the UK. No evidence of association was found between media peripherally related to TGD issues and referral rates.

**Conclusions and Relevance:**

This study found evidence of an association between increasing media coverage of TGD-related topics and increasing numbers of young people presenting to gender clinics. It is possible that media coverage acts as a precipitant for young people to seek treatment at specialist gender services, which is consistent with clinical experiences in which TGD young people commonly identify the media as a helpful source of information and a trigger to seek assistance.

## Key Points

**Question:**

Is media coverage of transgender issues associated with referrals of transgender and gender diverse (TGD) children and adolescents to specialist gender services?

**Findings:**

In this serial cross-sectional study across an 8-year study period during which more than 5000 TGD young people were referred to 2 pediatric gender clinics in the UK and Australia, a significant association was found between weekly referral rates and the number of TGD-related items appearing within the local media 1 to 2 weeks beforehand, for the UK only in week 1 and for Australia only in week 2.

**Meaning:**

An increase in media coverage of TGD-related topics over recent years was associated with an increase in the number of TGD young people presenting to 2 gender clinics on opposite sides of the world.

## Teaser

This cross-sectional study examines whether increases in transgender and gender diverse young people presenting to specialist gender clinics are associated with related media coverage.

## Introduction

Over the past decade, specialist gender identity clinics worldwide have witnessed a rapid increase in the number of transgender and gender diverse (TGD) young people being referred for clinical services.^1,2,3,4^ The underlying reasons for this increase are of great interest, but there is a lack of evidence to explain it. A common theory is that the improved recognition and acceptance of TGD people in society—coupled with greater public awareness of specialist gender services and available clinical interventions—are important. On the basis of the hypothesis that the media has played an influential role in increasing this recognition and awareness, this study sought to examine whether there was any association between local media stories about TGD young people and referrals to 2 different gender services, 1 in the UK and 1 in Australia.

Identity development is an important part of childhood and adolescence, and involves determining what it means to be a member of a relevant group, as well as trying to understand and question the expectations that society has regarding one’s group membership.^5,6,7^ Theories of identity development have suggested that most individuals go through a phase of identity questioning and searching before they achieve a relatively stable and committed identity. In our current digital climate, young people are exposed to an immense amount of information via traditional and digital media, which affect their health and well-being.^8^ Consistent with this, media have been shown to be an increasingly important influence on young people’s identity development, particularly in Western society.^9,10^

A critical aspect of identity is gender identity, which is an individual’s deep and inner feelings of belonging to either a specific binary gender (male or female) or identifying somewhere along or outside of the gender spectrum. Gender identity development is currently understood as a complex interplay of biological, psychological, and sociocultural factors.^11,12,13,14^ In the same way that media have been shown to be influential in other aspects of young people’s identity development, it seems plausible that media also play a role in gender identity development.^9,10^ Anecdotally, our experience working as clinicians with TGD youth certainly supports this, with many young people identifying specific media items as both a means by which they became aware of other TGD individuals as well as a prompt to explore their often covert yet long-standing feelings of gender diversity. To examine the possibility that media act as a catalyst for TGD young people to seek out specialist gender support, the aim of this study was to determine to what extent, if any, the increase in referrals rates to specialist gender services in the UK and Australia has been associated with relevant media coverage.

## Methods

This study was approved by the Royal Children’s Hospital human research ethics committee, which included a waiver of informed consent because it involved the secondary use of medical records.

### Clinical Setting, Referrals, and Study Population

Data on new patient referrals, which are usually initiated by primary care physicians, were obtained from 2 different specialist child and adolescent gender clinics: the Royal Children’s Hospital Gender Service (RCHGS) in Melbourne, Australia, and the Gender Identity Development Service (GIDS) in London, UK. Both clinics accept referrals of young TGD people up to the age of 18 years, and share a number of common features, including (1) offering a multidisciplinary service that includes not only mental health professionals but also medical and other allied health staff; (2) being publicly funded, which ensures that patients and their families do not incur any direct financial costs in attending; and (3) providing the only specialist service of its type in their relevant referral catchment areas, which is the state of Victoria (population, 6 million people) in the case of RCHGS and the entirety of England, Wales, Northern Ireland, and Scotland (population, 66 million) in the case of the GIDS, which offers a range of satellite and outreach clinics across the UK. Referral information, including the date of referral, age at referral, and assigned sex at birth, was obtained directly from the clinical databases and records of the RCHGS and GIDS for the period spanning January 1, 2009, to December 31, 2016.

### Media Coverage

After assessing a variety of commercial and publicly available media analysis tools, Google News (Alphabet) was chosen to extract relevant local media items from newspapers, online news sources, and television programs. Similar search strategies were implemented to obtain relevant media from Australia and UK, with minor changes made to identify media items that specifically mentioned each service or prominent clinicians from each service known to have appeared in the media. The search strategy used for the Australian media was as follows: (“transgender” OR “gender dysphoria” OR “gender identity disorder” OR “gender service”) AND ([“royal children’s hospital” OR “RCH” OR “michelle telfer” OR “campbell paul”] OR [“child” OR “adolescent”]) location:Australia. In contrast, the search strategy for the UK media was as follows: (“transgender” OR “gender dysphoria” OR “gender identity disorder” OR “gender identity development service” OR “GIDS”) AND ([“tavistock centre” OR “tavistock clinic” OR “tavistock and portman” OR “polly carmichael”] OR [“child” OR “adolescent”]) location:UK. Searches were conducted across the relevant referral period from January 1, 2009, to December 31, 2016. After obtaining the search results, individual media items were manually screened to confirm their relevance to TGD individuals, obtain their date of publication, and categorize them according to the nature of the media story. Specifically, media items were classified by a single coder (D.C.) as (1) being only peripherally related (eg, an article on Angelina Jolie that mentioned her transgender son in passing), (2) predominantly focused on TGD issues, or (3) directly mentioning GIDS or RCHGS.

### Statistical Analyses

Excel version 16 (Microsoft Corp) was used for data management, and Stata statistical software version 15 (StataCorp) and Prism statistical software version 7 (GraphPad) were used for statistical analyses. Statistical significance was set at *P* < .05 (2-sided tests). Basic descriptive analyses of participant characteristics (eg, age) were presented as median with interquartile range. Correlation between annual referral and media item numbers was undertaken using a Spearman rank correlation analysis.

To assess the possible association of media stories with referral numbers, media and referral data were first subdivided into weekly blocks across the 8-year study period for both RCHGS and GIDS. Fractional polynomial regression analysis was then performed to determine the association between the total number of media items appearing in a given week (the independent variable) and the number of referrals (the dependent variable) occurring either in that same week (week 1) or either of the 2 subsequent weeks (weeks 2 and 3), with separate regression analyses conducted for weeks 1, 2, and 3. Fractional polynomial regression modeling was specifically chosen to adjust for the effect of time, given the nonlinear increase in referrals and media items over time (Figure) and previous literature.^15,16^ The best fitting fractional polynomial for the regression modeling (which, in this case, was a third order polynomial) was selected on the basis of the model selection procedure recommend by Ambler and Royston^17^ using deviance (−2 × log likelihood). Additional fractional polynomial regression analyses were performed to determine the association between the different types of media items appearing in a given week and the number of referrals occurring in weeks 1, 2, or 3. Data analysis was performed in April 2019.

**Figure.**
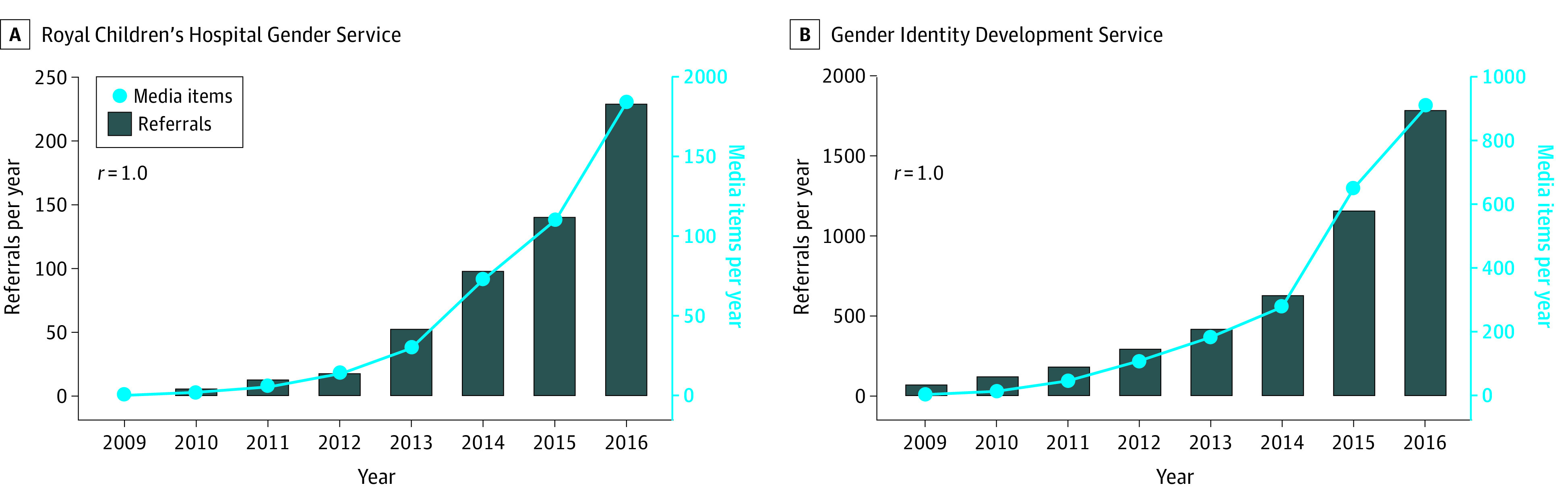
Annual Referrals to the Royal Children’s Hospital Gender Service and Gender Identity Development Service and Transgender- and Gender Diverse–Related Media Stories A, The 558 new referrals to the Royal Children’s Hospital Gender Service between 2009 and 2016 were positively correlated with the 420 stories appearing in Australian media over the same period (Spearman *r* = 1.0; *P* < .001). B, The 4684 new referrals to Gender Identity Development Service between 2009 and 2016 were positively correlated with the 2194 stories appearing in UK media over the same period (Spearman *r* = 1.0; *P* <  .001).

## Results

From 2009 to 2016, the total number of referrals was 5242: 4684 patients at GIDS, of whom 1847 patients (39.4%) were assigned male at birth and 2837 patients (60.6%) were assigned female at birth, and 558 patients at RCHGS, of whom 250 patients (44.8%) were assigned male at birth and 308 patients (55.2%) were assigned female at birth. A description of the patient demographic characteristics from each service is shown in Table 1. The total of number of media items was 2614, including 420 in Australia and 2194 in the UK. During this period, the annual number of referrals and TGD-related media items both rose sharply and showed a significant positive correlation (Spearman *r* = 1.0 and *P* < .001 for both RCHGS and GIDS) (Figure).

**Table 1.** Demographic Profile of Patients Referred to the Royal Children’s Hospital Gender Service and Gender Identity Development Service Between 2009 and 2016

Gender service	Assigned male at birth	Assigned female at birth
Royal Children’s Hospital Gender Service (n = 558)		
Patients, No. (%)	250 (44.8)	308 (55.2)
Age at referral, median (IQR), y	11.9 (7.0-15.5)	14.4 (12.7-15.8)
Gender Identity Development Service (n = 4684)		
Patients, No. (%)	1847 (39.4)	2837 (60.6)
Age at referral, median (IQR), y	15.3 (12.5-16.5)	15.4 (14.0-16.4)

Abbreviation: IQR, interquartile range.

To examine the association between referrals and media more closely, we performed nonlinear regression modeling on week-to-week data and observed that, for both the RCHGS and GIDS, referral numbers showed evidence of association with the total number of media items (Table 2). In the case of the RCHGS, the association with the total number of media items was most readily apparent when looking at referrals in week 2 (β̂ = 0.12; 95% CI, 0.04 to 0.20; *P* = .003). There was no evidence of association for referrals that occurred in week 1 (β̂ = 0.07; 95% CI, −0.01 to 0.15; *P* = .09) or week 3 (β̂ = 0.03; 95% −0.05 to 0.11; *P*  = .43). In the case of the GIDS, the total number of media items showed evidence of an association with referrals occurring in week 1 (β̂ = 0.16; 95% CI, 0.03 to 0.29; *P* = .01).

**Table 2.** Results From Nonlinear Regression Modeling of Media Items and Weekly Referrals for Each Gender Service

Referral wk	Gender service			
	RCHGS		GIDS	
	β̂ (95% CI)^a^	*P* value	β̂ (95% CI)^a^	*P* value
1^b^	0.07 (−0.01 to 0.15)	.09	0.16 (0.03 to 0.29)	.01
2^c^	0.12 (0.04 to 0.20)	.003	0.08 (−0.05 to 0.21)	.22
3^d^	0.03 (−0.05 to 0.11)	.43	0.10 (−0.03 to 0.23)	.14

Abbreviations: GIDS, Gender Identity Development Service; RCHGS, Royal Children’s Hospital Gender Service.

^a^ Adjusted for time, which showed a significant association with referrals in each model.

^b^ Referral in week 1 = β_0_ + β_1_ × media items in week 1 + β_2_ × time.

^c^ Referral in week 2 = β_0_ + β_1_ × media items in week 1 + β_2_ × time.

^d^ Referral in week 3 = β_0_ + β_1_ × media items in week 1 + β_2_ × time.

Additional nonlinear regression analysis was performed to determine whether there was any evidence of an association between the different types of media items appearing in a given week and the number of referrals occurring in weeks 1, 2, or 3 (Table 3). In the case of media stories that were only peripherally related to TGD issues, there was no evidence of an association with the number of referrals at either the RCHGS or GIDS (Table 3). In contrast, media items that specifically mentioned GIDS showed evidence of an association with referrals that occurred in week 1 (β̂ = 0.50; 95% CI, 0.11 to 0.88; *P* = .01) or week 2 (β̂ = 0.57; 95% CI, 0.18 to 0.98; *P* = .004) but not for referrals in week 3 (β̂ = 0.21; 95% CI, −0.18 to 0.60; *P* = .30). Meanwhile, media items that specifically mentioned the RCHGS showed no evidence of association with any of the weekly referral numbers (Table 3). Finally, there was evidence of an association between media items predominantly focused on TGD and weekly referral numbers at both the RCHGS and GIDS. Specifically, TGD-focused stories were associated with RCHGS referral numbers at week 1 (β̂ = 0.16; 95% CI, 0.04 to 0.28; *P* = .007) and week 2 (β̂ = 0.23; 95% CI, 0.11 to 0.35; *P* < .001) and with GIDS referral numbers at week 1 (β̂ = 0.22; 95% CI, 0.01 to 0.44; *P* = .04).

**Table 3.** Results From Nonlinear Regression Modeling of Different Types of Media Items and Weekly Referrals for Each Gender Service

Variable	Gender service			
	RCHGS		GIDS	
	β̂ (95% CI)^a^	*P* value	β̂ (95% CI)^a^	*P* value
Peripherally related media items in week 1, referral wk				
1^b^	−0.17 (−0.42 to −0.07)	.17	0.03 (−0.23 to 0.18)	.78
2^c^	−0.10 (−0.35 to 0.14)	.42	0.03 (−0.18 to 0.23)	.80
3^d^	0.10 (−0.15 to 0.35)	.44	0.01 (−0.20 to 0.22)	.91
TGD-focused media items in week 1, referral wk				
1^e^	0.16 (0.04 to 0.28)	.007	0.22 (0.01 to 0.44)	.04
2^f^	0.23 (0.11 to 0.35)	<.001	−0.04 (−0.25 to 0.18)	.75
3^g^	0.10 (−0.02 to 0.22)	.10	0.14 (−0.08 to 0.36)	.22
Media items in week 1 that mentioned RCHGS/GIDS, referral wk				
1^h^	−0.06 (−0.28 to 0.17)	.62	0.50 (0.11 to 0.88)	.01
2^i^	−0.07 (−0.29 to 0.16)	.57	0.57 (0.18 to 0.96)	.004
3^j^	−0.17 (−0.40 to 0.05)	.13	0.21 (−0.18 to 0.60)	.30

Abbreviations: GIDS, Gender Identity Development Service; RCHGS, Royal Children’s Hospital Gender Service; TGD, transgender and gender diverse.

^a^ Adjusted for time, which showed a significant association with referrals in each model.

^b^ Referral in week 1 = β_0_ + β_1_ × peripherally related media items in week 1 + β_2_ × time.

^c^ Referral in week 2 = β_0_ + β_1_ × peripherally related media items in week 1 + β_2_ × time.

^d^ Referral in week 3 = β_0_ + β_1_ × peripherally related media items in week 1 + β_2_ × time.

^e^ Referral in week 1 = β_0_ + β_1_ × TGD-focused medial items in week 1 + β_2_ × time.

^f^ Referral in week 2 = β_0_ + β_1_ × TGD-focused medial items in week 1 + β_2_ × time.

^g^ Referral in week 3 = β_0_ + β_1_ × TGD-focused medial items in week 1 + β_2_ × time.

^h^ Referral in week 1 = β_0_ + β_1_ × medial items in week 1 that mentioned RCHGS/GIDS + β_2_ × time.

^i^ Referral in week 2 = β_0_ + β_1_ × medial items in week 1 that mentioned RCHGS/GIDS + β_2_ × time.

^j^ Referral in week 3 = β_0_ + β_1_ × medial items in week 1 that mentioned RCHGS/GIDS + β_2_ × time.

## Discussion

As demonstrated by this cross-sectional study as well as by previous studies,^1,2,3,4^ there has been an increase in referrals for pediatric transgender health care over the past decade. Such a rapid increase in clinical demand is unusual within medicine, and the underlying reasons for this increase are of great interest. In this study, we observed evidence of an association between TGD-related media stories and referrals to 2 independent pediatric gender services on opposite sides of the world. Although our data do not provide evidence of causation, the results are nonetheless consistent with our own clinical experiences in which TGD patients commonly identify the media as a precipitant for them to seek clinical assistance. Moreover, our observation that relevant media stories showed an association with referrals within the first 2 weeks but not the third week suggests a model in which media items have a short-lived, antecedent effect. Taken together, our results support the hypothesis that media can help to promote referrals, and there are several ways in which media might help to do so.

First, media serve as an important means by which young people explore, recognize, and understand their overall identity, and it seems reasonable that the same might also be true of gender identity more specifically.^18,19,20^ Anecdotally, many of our patients have reported that their clandestine feelings of gender diversity were brought to the surface by media stories that prominently featured TGD individuals and helped them to appreciate that others share similar feelings. Our observation that media stories that focus on TGD issues, but not those peripherally related, were significantly associated with referral numbers at the RCHGS and GIDS accords with this idea. It is also consistent with previous studies that have shown that, although some young people have good access to relevant knowledge within their immediate environment (eg, via family, peers, or school), for many others, media provide information that is otherwise missing from their lives.^21,22^ This might include young people obtaining relevant exemplars from media to help them understand their developing identity or explicitly selecting media content to explore and construct their identities.^9,18,23,24^

Second, media stories that raise awareness of relevant clinical services could act as a direct impetus for young people or their families to obtain a referral. Consistent with this, we observed that news items that mentioned GIDS were associated with an increase in the number of referrals to this service. Media stories related to clinical gender services could serve several functions. For instance, these items might empower young TGD people to initiate a referral through their general practitioner either by alerting the young person (or their families) to the existence of local clinical services and/or by promoting knowledge of specific treatment options (eg, hormone therapy). Second, by providing young TGD people with the realization that professional services and clinical pathways exist to assist them, media items might help to validate and legitimize their experiences and thus counteract the disbelief and nonaffirmation from others that many of them face after coming out.

Third, it is likely that TGD-related media has improved the recognition and acceptance of gender diversity in wider society, and this may have helped to create an environment that fosters referrals. For example, the increasing media portrayal of TGD individuals not only in real life (eg, Caitlyn Jenner and Chelsea Manning) but also in popular fiction (eg, in television shows, such as *Transparent* and *Orange Is the New Black*) is likely to have helped not only create an incremental shift in public awareness but also normalize gender diversity.^25^ Such changes are likely to have occurred gradually over time and might be one reason why time itself was significantly associated with referral numbers.

Of note, it is also possible that the association between TGD-related media and referrals exists indirectly and arises not on account of the media promoting referrals but for alternative reasons. For example, recent shifts in societal attitudes toward TGD individuals might have acted as the primary driver for both greater media attention and increased referrals, causing both to increase simultaneously. Nevertheless, consistent with our findings, previous studies have reported that the media can promote health care utilization across different settings, including increases in immunization uptake^26^ as well as specific forms of cancer treatment.^27^

There are several other areas that would be interesting to explore via future research. First, given the increase in referrals in recent years, it would be helpful to determine how long this trend is likely to continue to allow services to better plan for future demand. If media have been important in promoting referrals via knowledge transfer of TGD issues, then one would expect there to be a saturation point at which awareness and acceptance of these issues will plateau within the community, with referrals eventually reaching a steady state. Second, it would be interesting to examine whether media stories are also associated with referrals of particular patient subgroups. For instance, a consistent observation over the past 2 decades has been a shift in the ratio of birth-assigned male and female patients presenting to pediatric gender clinics.^1,2,3,4,28^ Specifically, there was a preponderance of patients who were assigned male at birth in the past, but in recent years, patients who were assigned female at birth have come to represent most referrals for reasons that are entirely unclear. Knowing whether media stories are associated with referrals based on birth-assigned sex might provide some insights into this new development. Another recently observed change in referral patterns has been the increased presentation of TGD young people with a nonbinary (ie, neither exclusively male or female) gender identity, and it would be helpful to know whether media items about nonbinary identities have been associated with this change.^29^ Finally, determining whether the sentiment and tone of media stories affect the association between media and clinical referral numbers would be important. For example, in the past few years, there has been a significant amount of negative press coverage of the GIDS in the UK, and it is possible that this may have dissuaded some young people and their families from seeking care. Testing whether negative media coverage is associated with reduced referral rates (and conversely whether positive coverage is associated with increased referrals rates) would thus be a useful next step.

However, we are also mindful that others have speculated that increased media content (specifically via social media) might act as a double-edged sword or a means of social contagion, whereby some individuals erroneously come to believe through exposure to such media that their nonspecific emotional or bodily distress is due to gender dysphoria and being TGD.^30,31^ Here, the implied outcome is that such individuals will then access gender-related medical interventions and eventually come to regret these once they realize that they are in fact not TGD. Historically, rates of regret are very low among patients attending relevant gender services. For example, among almost 4000 patients who attended the VU University Gender Clinic in the Netherlands between 1972 and 2015 and received medical treatment, only approximately 0.5% were identified as having regret, and there was no evidence that regret rates had increased over time.^4^ Nevertheless, as clinicians working in this field, we are highly mindful of the risk of regret throughout the assessment and treatment process, and it will be important to continue not only to provide adequate counselling before such interventions are undertaken but also to observe whether regret rates increase in the face of greater media attention and more referrals.

### Limitations

This study has limitations. Our data provide evidence of an association between relevant media stories and clinical referrals of TGD young people but, given the nature of the study design, no indication of causation. Moreover, our study weighted each media item equally (despite likely differences in reach and accessibility) and was unable to quantify actual levels of media exposure among referred patients. Another limitation is that this association might not generalize to other services. After all, the RCHGS and GIDS were chosen for this study because they are publicly funded, do not charge attendance fees, and provide the only specialist pediatric gender services within their respective regions, thus ensuring that their referral data are likely to be relatively comprehensive and complete accounts of clinical demand within each catchment area; most other pediatric gender clinics will not share these same characteristics. Another important limitation of our study is that it only examines traditional forms of media and does not include social media, which are a very important source of information as well as a critical means for finding support and fostering connectedness and community among young people, including TGD adolescents.^32,33,34,35^ Social media were not examined in our study given the difficulties of readily accessing such information across time, but in the future, collaborations with relevant social media companies, such as Facebook, might allow us to address this gap.

## Conclusions

This cross-sectional study found evidence of an association between media coverage of TGD issues and presentation of young people to gender clinics in Australia and the UK. These findings accord with our clinical experiences, which suggest that relevant media stories may help to empower young TGD people and their families to appropriately seek clinical care.
